# Supplementary material for: Genome-Wide Association Study on Reproduction-Related Body-Shape Traits of Chinese Holstein Cows
Source: Animals (Basel). 2021 Jun 28;11(7):1927. doi: 10.3390/ani11071927 (PMC8300307; doi:10.3390/ani11071927)
Supplement: Supplementary file 1 [file animals-11-01927-s001.zip › animals-1191985-supplementary/supplementary/Figure S1 Phenotype distributions and correlations among LS, RA, and PW across the farm.pdf]

Supplementary material

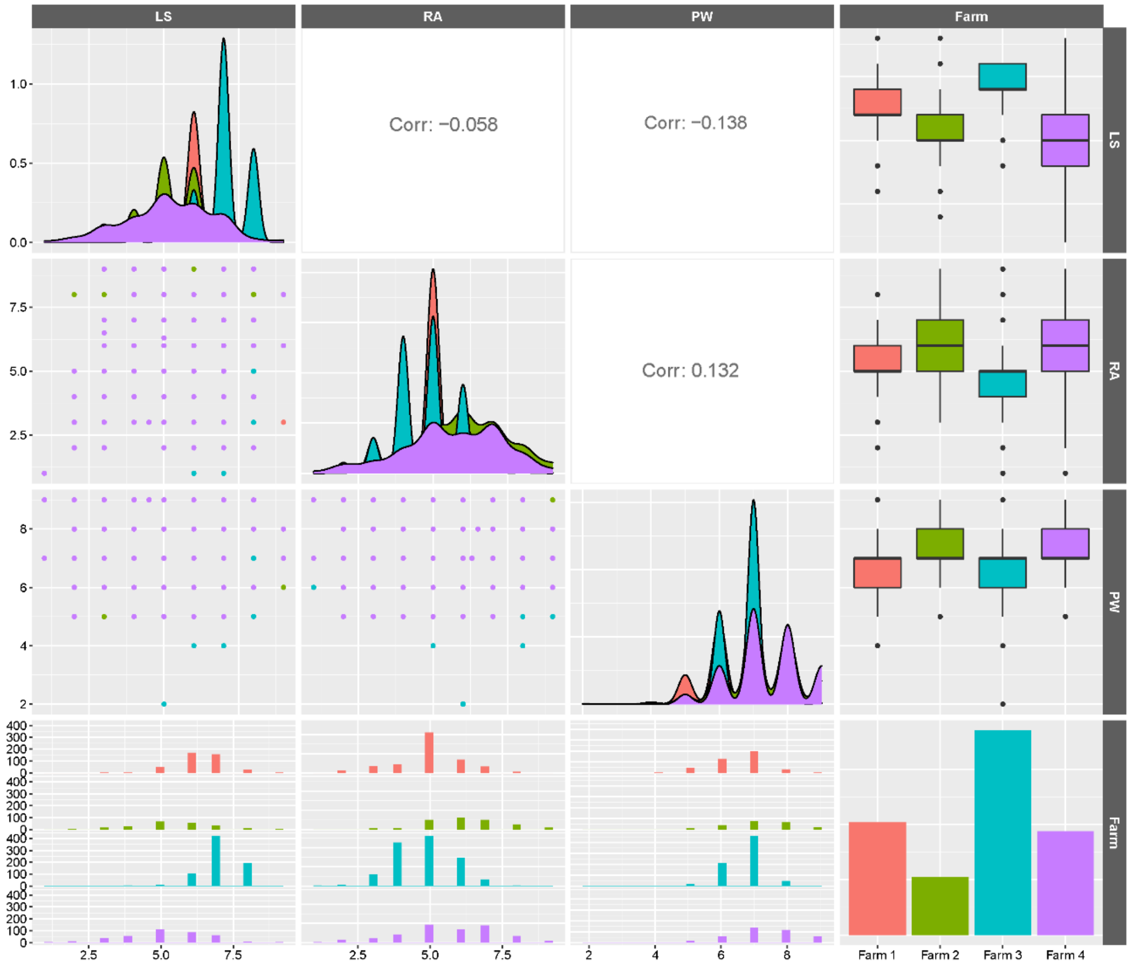

Figure S1. The phenotype distributions and correlations among the 3 body-type traits of 1730 cows across the 4 farms. The traits include Loin Strength (LS), Rump Angle (RA), and Pin Width (PW). The distributions of values are displayed on the diagonals and the correlations are illustrated as scatter plots off the diagonals.
