# Supplementary material for: Genome-Wide Association Study on Reproduction-Related Body-Shape Traits of Chinese Holstein Cows
Source: Animals (Basel). 2021 Jun 28;11(7):1927. doi: 10.3390/ani11071927 (PMC8300307; doi:10.3390/ani11071927)
Supplement: Supplementary file 1 [file animals-11-01927-s001.zip › animals-1191985-supplementary/supplementary/Table S2 Phenotypic and genetic correlations between LS, RA and PW of cows.pdf]

Table S2. Phenotypic and genetic correlations between LS, RA and PW of cows

Trait	LS	RA	PW
LS		-0.06	-0.14
RA	0.36 (0.17)		0.13
PW	-0.08 (0.03)	0.27 (0.08)	

Note: LS: Loin Strength; PS: Pin Setting; PW: Rump Angle. Above the diagonal are phenotypic correlations, below the diagonal are genetic correlations and in parentheses are standard errors about genetic correlations.
