# Supplementary material for: Genome-Wide Association Study on Reproduction-Related Body-Shape Traits of Chinese Holstein Cows
Source: Animals (Basel). 2021 Jun 28;11(7):1927. doi: 10.3390/ani11071927 (PMC8300307; doi:10.3390/ani11071927)
Supplement: Supplementary file 1 [file animals-11-01927-s001.zip › animals-1191985-supplementary/supplementary/Table S3 Candidate genes within 200 Kb from the significant SNPs identified in the genome-wide association studies for LS, RA, and PW.pdf]

Table S3. Candidate genes within 200 Kb from the significant SNPs identified in the genome-wide association studies for LS, RA, and PW

Traits	SNPs	Genes	Entrezid	CHR	Location
LS	rs42946768	CDH12	540672	20	51192127-51567420
	rs109073659	PCDH9	509935	12	39460262-40603455
	rs133475777	DTHD1	100140203	6	55682987-55761611
		ARAP2	512010	6	55443414-55641572
		LOC112447062	112447062	6	55676300-55680617
		DTHD1	100140203	6	55682987-55761611
		LOC104972734	104972734	6	55963434-56027535
	rs43162548	TARP	100335800	4	50141812-50162835
		LOC112446379	112446379	4	50123266-50126142
		LOC112446378	112446378	4	50142032-50148466
		CTTNBP2	530341	4	50482582-50651615
RA	rs43486059	LOC781835	781835	6	102563753-102607151
		HSD17B13	618192	6	102192495-102212283
		HSD17B11	527592	6	102227494-102276972
		NUDT9	517589	6	102320425-102357772
		SPARCL1	507537	6	102370630-102423199
		LOC104968931	104968931	6	102435304-102453699
		DSPP	100337125	6	102495020-102501526
		DMP1	281118	6	102526308-102543091
		LOC523503	523503	6	102631253-102676741
		PPP2R2C	782110	6	102716696-102883585
		WFS1	100298456	6	102896920-102928905
	rs137244035	LOC112447415	112447415	7	45062806-45065666
		LOC112447416	112447416	7	45263769-45274033
	rs43366267	SH3BP4	520462	3	114535121-114633955
		LOC112445894	112445894	3	114482144-114490858
		LOC112446038	112446038	3	115011161-115024686
		AGAP1	522241	3	115044734-115605268
	rs43352090	ATG4C	531455	3	82756137-82888314
		ITGB3BP	614469	3	82052235-82157908
		ALG6	510138	3	82149562-82208246
		FOXD3	532383	3	82243110-82245982
		LOC101906485	101906485	3	82246130-82248189
		LOC112446160	112446160	3	82787128-82787234
PW	rs109578471	USP6NL	530462	13	12568065-12779550
		UPF2	781181	13	12301048-12396488
		LOC101907256	101907256	13	12402359-12403416
		PROSER2	781700	13	12436965-12492165
		ECHDC3	617368	13	12494254-12533520
		LOC112449248	112449248	13	12766847-12771138

		LOC104973708	104973708	13	12788585-12799155
		LOC112449249	112449249	13	12803905-12808553
		CELF2	777790	13	12976707-13184863
	rs43430205	CNTN3	526697	22	27019078-27418487
	rs42051017	LOC101907665	101907665	29	3661815-3683578

Note: LS: Loin Strength; PS: Pin Setting; PW: Rump Angle; CHR: Chromosome
